# Next-generation sequencing identified a novel *CACNA1A* I1379F variant in a familial hemiplegic migraine type 1 pedigree: A case report

**DOI:** 10.1097/MD.0000000000028141

**Published:** 2021-12-23

**Authors:** Huiyan Luan, Lei Zhang, Sijin Zhang, Meng Zhang

**Affiliations:** a Department of Neurology, China-Japan Union Hospital of Jilin University, Changchun, Jilin, China; b Department of Pediatrics, the Second Hospital of Jilin University, Changchun, Jilin, China.

**Keywords:** *CACNA1A*, familial hemiplegic migraine, genetics, mutation, whole-exome sequencing

## Abstract

**Rationale:**

Familial hemiplegic migraine (FHM) is a rare, autosomal dominant migraine with aura. *CACNA1A* encodes the α1A subunit of P/Q-type voltage-gated calcium channels, and its mutations have been associated with a wide spectrum of episodic and chronic neurological disorders, including FHM type 1 (FHM1).

**Patient concerns:**

A Chinese girl and some of her relatives who presented with hemiplegia with or without migraine were found to carry a novel heterozygous missense variant, I1379F, in *CACNA1A* by whole-exome sequencing. The variant consegregated with the disease and was predicted to be pathogenic.

**Diagnosis:**

The patient was diagnosed with FHM1 clinically and genetically.

**Interventions:**

Prophylactic therapy with flunarizine 5 mg daily was prescribed to the patient.

**Outcomes:**

Therapy with flunarizine was terminated after a few weeks. The intensity of the attacks was the same as before.

**Lessons:**

This case indicates that FHM should be considered when a patient manifests with episodic hemiplegia without migraine. In addition, genetic testing is an indispensable method to identify atypical attacks of hemiplegic migraine.

## 1 Introduction

Hemiplegic migraine (HM) is a rare type of migraine with aura whose attacks are associated with different degrees of hemiparesis. It may also present sensory, visual, or language impairments during aura. However, attacks presenting with only hemiparesis, but without migraine, are scarcely reported. Familial hemiplegic migraine (FHM, MIM#141500) is an autosomal dominant subtype of HM^[1,2]^ and is genetically heterogeneous. Mutations in these 3 genes are now known to be associated with FHM: *CACNA1A*, *ATP1A2*, and *SCNA1*. Recent research has indicated that *PRRT2* is the fourth FHM gene.^[3]^ Here, we report a novel heterozygous *CACNA1A* variant, I1379F, associated with FHM type 1 (FHM1) in a Chinese family who sometimes presented with only hemiparesis. This research indicates that genetic testing is an indispensable method for identifying atypical HM attacks.

## 2 Clinical data

**Figure 1:**
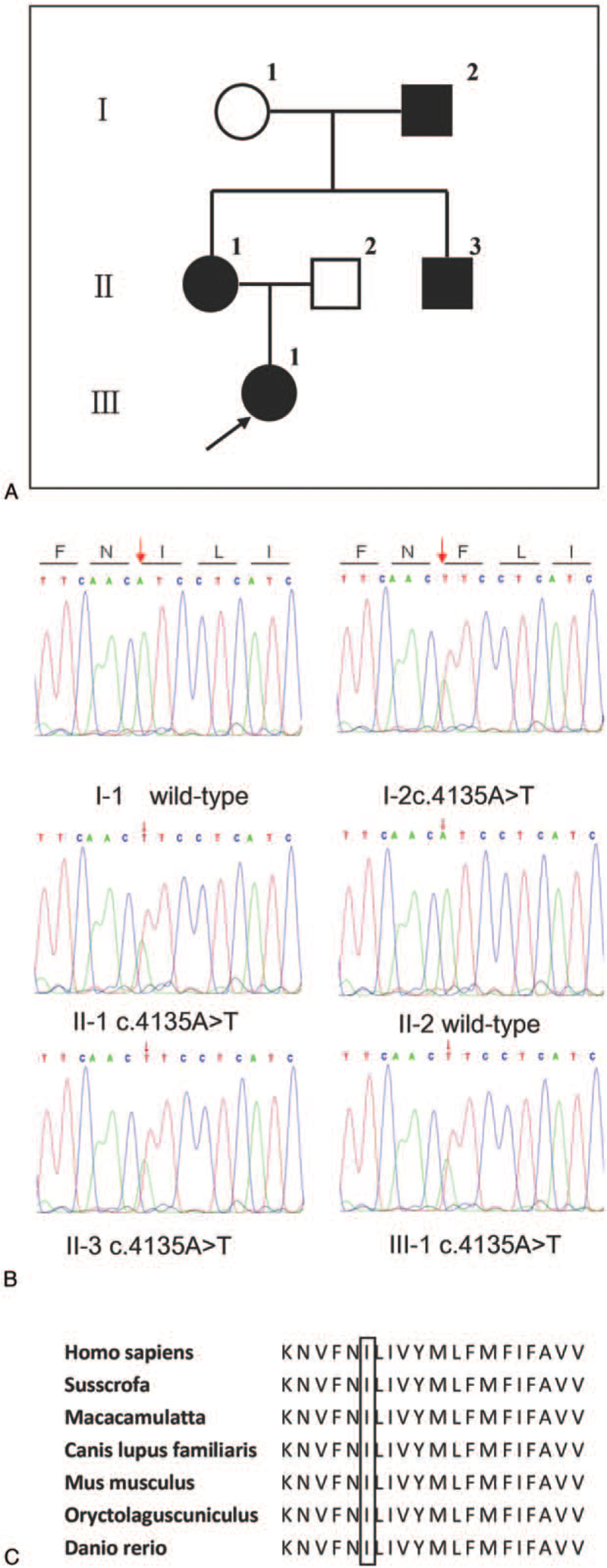
Detection of I1379F *CACNA1A* in the Chinese family with FHM1. (A) Pedigree of the family with FHM1. The proband (III-1) is indicated by an arrow. Filled symbols, individuals with HM; open symbols, unaffected individuals; squares, male; circles, female individual. (B) A heterozygous A-to-T transition at the 4135 nucleotide was confirmed in the proband and her mother, grandfather and uncle by Sanger DNA sequencing and cosegregated with HM. The c.4135 A>T variant produces an amino acid change from isoleucine to phenylalanine at codon 1379 (I1379F). Arrow denotes the variant. (C) The isoleucine in this position is highly conserved across several species.

The proband (III-1 in Fig. 1A) was an 11-year-old girl in 2019 when she was referred to the hospital. Her first attack occurred at the age of 9 years, and she had 7 to 8 attacks. Usually, the first sign of the attack is the feeling of being a little annoyed without any cause. At this stage, if she tried her best to relax, the attack could sometimes cease. Then, flickering diamonds or decomposable objects, such as a person without a head or legs, wobbled in her vision. A few minutes later, she experienced hemilateral weakness involving the face and limbs of the same side, lasting for 10 to 20 minutes. The symptoms vanished after rest for 20 or 30 minutes. In only 2 episodes, a slightly throbbing headache appeared. When the attack happened in public, she was very embarrassed and shamed about it. There was no obvious trigger for the attacks. She had never experienced ictal and interictal gait unsteadiness, vertigo, or related nausea or vomiting. She had undergone many examinations, including brain magnetic resonance imaging (MRI), electroencephalogram, and cerebral computed tomographic angiography, but did not have a definite diagnosis.

### 2.1 Family history

Her mother, grandfather, and uncle (II-1, I-2 and II-3 in Fig. 1A) had similar attacks. Her mother also often felt angry before the attack. If she tried to relax or rest, the attack might have ceased. Emotional stress, sleep deprivation, alcohol, and caffeine was some triggers for her attacks. Migraine following hemiparesis was also very scarce. She had never had gait unsteadiness or vertigo. The frequency of attacks was 8 to 10 times per year. She underwent brain MRI, but without positive findings.

### 2.2 Physical examination and auxiliary examinations

Examination of the proband on admission was unremarkable. Laboratory investigations, brain MRI, and electroencephalogram showed a normal pattern.

### 2.3 Genetic testing

DNA was extracted from blood samples from 4 patients and 2 unaffected relatives of this family. Whole-exome sequencing identified a novel heterozygous c.4135A>T variant in exon 26 of *CACNA1A* (NM_001127222.2). This leads to the substitution of isoleucine with phenylalanine at codon 1379 of *CACNA1A* (I1379F). The variant cosegregated with the disease in this family (Fig. 1B). Isoleucine in this position was highly conserved (Fig. 1C). It is not present in ExAC and is predicted “deleterious” and “possibly damaging” by SIFT and PolyPhen-2 respectively. The patient was diagnosed with FHM1 clinically and genetically.

### 2.4 Treatment and outcome

Prophylactic therapy with flunarizine 5 mg daily was prescribed to the proband but terminated after a few weeks because of the scarcity of attacks. The intensity of the attacks was the same as before. Her mother started treatment with flunarizine 10 mg daily, and no further hemiplegic episodes have been reported to date.

## 3 Discussion

Pathogenic mutations in *CACNA1A* lead to autosomal dominant episodic disorders, including episodic ataxia type 2 (EA2), spinocerebellar ataxia type 6 (SCA6), FHM1, and developmental and epileptic encephalopathy 42.^[4]^ FHM1 is characterized by the occurrence of motor deficits during the aura. EA2 clinically presents with paroxysmal attacks of generalized ataxia associated with nausea, vomiting, vertigo, diplopia, nystagmus, dysarthria, and tinnitus. Conversely, SCA6 is a chronic disorder with adult-onset moderate to severe permanent and progressive ataxia, dysarthria, and oculomotor disturbances. The phenotype of developemental and epileptic encephalopathy 42 includes intellectual disability, attention-deficit hyperactivity, autism, nystagmus, and intermittent ataxia.^[4]^ Clinical overlap between these disorder symptoms has been reported frequently, especially in EA2 and FHM1. One-third of FHM1 patients experienced ictal gait ataxia and dysarthria, and 50% of EA2 patients had migraine during the attacks or occurred independently.^[4]^ At the same time, 1 *CACNA1A* mutation might cause overlapping features, including FHM, epileptic encephalopathy, and EA2 to SCA6 among members of the same family.^[5]^ In this study, all 4 patients carrying *CACNA1A* I1379F complained of hemiparesis with or without migraine and had never presented with ataxia or encephalopathy. In addition, another unique presentation of this pedigree is that the attack could be stopped if the patient tried to relax, and it is a feature that has never been reported to our knowledge. More research and observations are necessary to elucidate the clinical characteristics of this novel variant.

*CACNA1A* encodes the poreforming CaVα1 subunit of P/Q-type (Cav2.1) calcium channels, which are situated in the presynaptic terminals of neurons. P- and Q-types are highly expressed in Purkinje cells and cerebellar granule neurons. Cav2.1 channels mediat the entry of Ca^2+^ ions into excitable cells and are involved in a variety of Ca^2+^-dependent processes, such as muscle contraction, neuronal excitability, hormone and neurotransmitter release, and gene expression.^[6,7]^ α1A subunit encoded by *CACNA1A* controls the voltage dependence, ionic selectivity, and major gating kinetics of P/Q-type channels. It consists of 24 transmembrane α-helical segments divided into 4 repeated domains (I–IV), which contain 6 integral transmembrane segments (S1–S6). Missense mutations of *CACNA1A,* which change the voltage-dependence of neuronal CaV2.1 channels toward more negative membrane potentials, have gain-of-function effects and are associated with FHM1. They lead to increased Ca^2+^ influx into the presynaptic terminal, enhanced neurotransmission, and neuronal hyperexcitability.^[8]^ Most pathogenic variants in *CACNA1A* are missense variants lying in significant functional domains of the calcium channel, that is, the voltage sensor, pore, and pore-lining loops. The closest pathogenic mutation to codon 1379 reported here was Y1384C. *CACNA1A* Y1384C carriers presented with mental retardation, progressive deteriorating chronic ataxia, prolonged HM episodes, and early cerebellar atrophy.^[9]^ Functional analysis of Y1384C showed a significant loss of function in current density and changes in gating properties in the neuronal Cav2.1 channel.^[10]^ In this Chinese family, *CACNA1A* I1379F was identified as the cause of pure FHM without ataxia or mental retardation. I1379F is a missense variant located in the transmembrane S5 segment of the third domain of Cav2.1, the same segment as Y1384C. The reason why these patients in this pedigree have a much milder presentation than Y1384C might be the conden of 1379 is less important and there is only a small physicochemical difference between isoleucine and phenylalanine. Another possibility is phenotypic heterogeneity in one genotype. More reports of I1379F patients are necessary to clarify the real spectra and help to establish the phenotype-genotype correlation.

When a patient presents with sudden onset of transient neurological deficits, the suspicion of vascular events or epilepsy is the first consideration, especially in this patient whose attacks sometimes presented with pure hemiplegia without migraine. HM should be considered after a detailed examination of these conditions. Indeed, this proband and her mother had undergone an extensive work-up to address their diagnosis but did not obtain a definitive result. In terms of the diagnostic approach, the precise definition of symptoms is pivotal. Genetic testing is beneficial. With atypical presentations of HM, 3 possible genes for FHM, and the large number of exons in *CACNA1A,* genetic diagnosis using conventional Sanger sequencing is extremely difficult. In contrast, next-generation sequencing approaches have opened the door for massive parallel sequencing of genes. It is of great help to reliably detect disease-causing mutations, as well as to find a large number of unclassified variants in different genes that might be related to the patient's phenotypes. Furthermore, it is helpful for detecting phenotypic and genotypic heterogeneity. The detection of a causal mutation in the setting of a paroxysmal disorder as HM provides not only a definite diagnosis but also a targeted and effective treatment.

In conclusion, the clinical features of FHM are complex and vary greatly. When a patient experiences episodic hemiplegia with or without migraine, the diagnosis of FHM should be considered after exclusion of common reasons. In addition, genetic analysis is helpful for doctors to judge the atypical manifestations and provide proper treatment to patients in a timely manner, which may improve the quality of life of patients.

## Author contributions

**Methodology:** Huiyan Luan.

**Supervision:** Meng Zhang.

**Writing – original draft:** Lei Zhang.

**Writing – review & editing:** Sijin Zhang.
